# Spectrum of prostatic lesions

**DOI:** 10.1186/1755-7682-6-36

**Published:** 2013-09-25

**Authors:** Hafiz Muhammad Aslam, Nazish Shahid, Naseem Ahmad Shaikh, Hiba Arshad Shaikh, Shafaq Saleem, Anum Mughal

**Affiliations:** 1 Department of Pathology, Dow Medical College, Dow University of Health Sciences, Karachi, Pakistan

**Keywords:** Prostatic hyperplasia, Prostatic carcinoma, Prostate

## Abstract

**Background:**

Prostate gland of male reproductive system is about the size of walnut and surrounds the urethra. Most frequently encountered diseases affecting prostate are Prostatitis, Benign prostatic hyperplasia and Prostatic cancer .Our objective of study was to evaluate the spectrum and correlation of prostatic lesions with presenting complaints of patient.

**Methods:**

It was a cross-sectional study conducted in Pathology Department of Dow Medical College, Dow University of Health Sciences during the period of 1^st^ January 2010 to December 2012. Pathology department of Dow Medical College collected specimens from both Civil Hospital and Lyari General Hospital Karachi, Pakistan. Specimens were taken through transurethral resection of prostate (TURP), simple prostatectomy and radical prostatectomy. A questionnaire was made and information including name, age, ward name of hospital, laboratory number, clinical diagnosis and symptoms were noted in it. Data was entered and analyzed through SPSS 19.

**Result:**

During the targeted months, 48 prostatic specimens were received with a mean age of 65.7 + -7.6 years. Common presenting complains were urinary retention in 23(47.9%) patients, followed by dribbling in 12(25%). Out of 48 patients, 42 have Benign Prostatic Hyperplasia and 6 have Prostatic Adenocarcinoma. Both Benign Prostatic Hyperplasia and Prostatic Adenocarcinoma were more prevalent in the age group of 60-70 years.

**Conclusion:**

Frequency of prostatic cancer is on the rise and measures should be taken for its early detection. Screening protocols and awareness programs need to be introduced. Screening programs should be focused on level of androgens and molecular pathogenesis.

## Introduction

Worldwide, diseases of Prostate gland are responsible for significant morbidity and mortality among adult males [1]. It is estimated that number of males in the U.S who will experience prostatitis during their lifetimes range up to 50% [2]. Prostate cancer is the most common malignant tumor in men all over the world and is also the second important cause of cancer related deaths in men after lung cancer. The American Cancer Society’s estimates for prostate cancer in the United States for 2013 are such that about 238,590 new cases of prostate cancer will be diagnosed and About 29,720 men will die of prostate cancer. It is estimate that about 1 man in 6 will be diagnosed with prostate cancer during his lifetime. It occurs mainly in older men and about 6 cases in 10 are diagnosed in men aged 65 or older. The average age at the time of diagnosis is about 67. Prostate cancer can be a serious disease, but most men diagnosed with prostate cancer do not die from it. In fact, more than 2.5 million men in the United States who have been diagnosed with prostate cancer at some point are still alive today [3]. In Pakistan, prostatic cancer was fifth commonest tumor occurring in 7.3% of all men [4].

Prostate gland of male reproductive system is about the size of walnut and surrounds the urethra. It produces and store a milky white fluid which becomes the part of semen and consists of sugars, proteins and other chemicals which help the sperm to survive in female genital tract [5]. It is a retroperitoneal organ encircling the neck of bladder and urethra and is devoid of distinct capsule. In adults, Prostatic parenchyma can be divided into four biological and anatomical zones: peripheral, central, transitional and anterior fibromuscular stroma. Most hyperplasia occurs in transitional zone while most carcinoma originates in the peripheral zone. Most frequently encountered diseases affecting prostate are Prostatitis, Benign prostatic hyperplasia and Prostatic cancer [1]. Inflammation of prostate gland is called Prostatitis, it is characterized by urinary frequency, dysuria, body aches and sometimes fever. Prostatitis may be infective and non-infective [5]. In some males, prostate enlargement occurs with the increase in age [6].

Benign Prostatic Hyperplasia or Nodular Hyperplasia is the non-malignant adenomatous overgrowth of prostate gland [7]. It is characterized by hyperplasia of prostatic stromal and epithelial cells, resulting in the formation of large discrete nodules in peri-urethral region of prostate [6]. It often presents with lower urinary tract symptoms [8]. Symptoms include weak stream, hesitancy, frequency, urgency, nocturia, incomplete emptying, terminal dribbling, overflow or urge incontinence and complete urinary retention [7]. Incidence of prostatic cancer is increases proportionally after age of 50 years [1]. In approximately, 70% of cases it arises in the peripheral zone of gland particularly in the posterior location [4]. Adenocarcinoma is its most common histological variant [1]. Most important risk factors for developing Prostate carcinoma are Family history, increasing age, lack of exercise and high calcium intake [9]. In most cases, it is asymptomatic and develops slowly. However, it may present with pain, difficulty in urinating and problems during sexual intercourse [5].

Our main goal of study was to evaluate the spectrum of prostatic disorders and assess the relationship of patient presenting complains with prostatic disorders.

## Methodology

### Study setting

It was a cross-sectional study conducted during the period of 1^st^ January 2010 to December 2012 in Pathology department of Dow Medical College, which collects specimens from both Civil Hospital and Lyari General Hospital Karachi, Pakistan. These are two of the largest public sector, tertiary care hospitals in Karachi. They provide subsidized healthcare to patients, majority of whom belong to low socio-economic class.

### Study protocol

Specimens examined were taken by transurethral resection of prostate (TURP), simple prostatectomy, radical prostatectomy and radical cystoprostatectomy. Biopsies were kept in 10% neutral buffered formalin. Specimens were grossly examined and size/quantity and weight of all specimens were recorded. Abnormalities such as increase in weight or size and gross characteristics such as nodular and cystic changes were noted. After cutting and processing of sections they were embedded in paraffin. Sections were 4-6 micrometer thick and they were stained with Hematoxylin & Eosin in all cases, while periodic acid Schiff (PAS) and mucicarmine were also used in some cases.

### Diagnostic criteria

#### Diagnostic criteria for prostatic cancer

##### Nuclear changes

Presence of prominent nucleoli was advocated as diagnostic criterion of prostate cancer. Most of these prominent nucleoli were in areas of inflammation, basal cell hyperplasia, atrophy, or Paneth cell-like change. In addition to nucleolar prominence, multiple nucleoli and nucleolar margination have also been suggested as diagnostic criteria for prostate cancer. Multiple nucleoli are never found in benign gland.

###### Perineural invasion

Presence of glands in a perineural location used to be considered as a diagnostic hallmark of malignancy. Circumferential growth or intraneural invasion should be regarded as pathognomonic of cancer.

###### Cytoplasmic features

Cytoplasmic features in malignancy vary from clear amphophilic to eosinophilic. They are very useful features in differentiation between benign and atypical/cancerous glands.

###### Collagenous micronodules

Collagenous micronodules are another recently described histological observation in Prostate cancer. These microscopic nodular aggregates of paucicellular eosinophilic fibrillar stroma are a specific, but infrequent, diagnostic clue in prostatic adenocarcinoma.

###### Intraluminal contents

Prostatic crystalloids are intraluminal, eosinophilic and refractile structures of varying size and shape, which are closely associated with prostate cancer. Presence of intraluminal acidic mucin also has been advocated as useful supportive evidence in the diagnosis of prostate adenocarcinoma [10].

### Diagnostic criteria for benign prostatic hyperplasia

Nodularity is the hallmark of Benign Prostatic Hyperplasia. In the usual case prostate enlarges up to 100gm and nodular hyperplasia of the prostate originates almost exclusively in the inner aspect of Prostate gland. On cross section, the nodules vary in color and consistency. In nodules that contain mostly glands, tissue is yellow pink with soft consistency and a milky white prostatic fluid oozes out of these areas. In nodules which are composed primarily of fibromuscular area, each nodule is pale gray, tough and does not exclude fluids [10].

### Questionnaire

A questionnaire was made and information including name, age, ward of hospital, laboratory number, clinical diagnosis and presenting complains were noted. Prostatic lesions were classified into benign and malignant. They were tabulated with age and symptoms.

### Ethical review

Research was ethically approved from the Institutional Review Board of Dow Medical College, Karachi which was valid for both Civil Hospital and Lyari General Hospital.

### Operational defination

#### Transurethral resection of prostate (turp)

Transurethral resection of the prostate, commonly known as a TURP is a urological operation. It is used to treat benign prostatic hyperplasia (BPH). As the name indicates, it is performed by visualizing the prostate through the urethra and removing tissue by electrocautery or sharp dissection. This procedure is done with spinal or general anesthetic. A triple lumen catheter is inserted through the urethra to irrigate and drain the bladder after the surgical procedure is complete.

### Radical prostectomy

Radical prostatectomy is surgery to remove all of the prostate gland and some of the tissue around it to treat prostate cancer.

### Statistical analysis

All data was entered and analyzed through SPSS 19. Mean and standard deviation were evaluated for continuous data. Frequency and percentage were calculated for categorical data.

## Result

During the period of three years from 1st January 2010 to 31st December 2012, 48 prostatic specimens were received. Age range of patients was 40-90 years. Mean age of presentation was 65.7 + -7.6 years. Majority of cases were in the age group 60 -70 years (58.3%) (Table 1).

**Table 1:** Represent pattern of prostatatic lesion and presenting complains of patients

**S.no**	**Variables**		**Frequency (n)**	**Percentage (%)**
1	**AGE**			
		a) 40-50	3	6.3
		b) 50-60	11	22.9
		c) 60-70	28	58.3
		d) 70-80	4	8.3
		e) 80-90	1	2.1
2	Diagnosis	a) Hyperplasia	42	87.5
		b) Carcinoma	6	12.5
3	**SYMPTOMS**			
		a) Micturition	10	20.8
		b) Burning	2	4.2
		c) Increase in frequency	5	10.4
		d) Increase in frequency and burning	3	6.3
		e) Abdominal pain	4	8.3
		f) Incontinence	8	16.7
		g) Inguinal swelling	8	16.7
		h) Urinary retention	23	47.9
		i) Dribbling hematuria	12	25

Patients were commonly present with urinary retention in 23(47.9%), followed by dribbling 12(25%), incontinence 8(16.7%) and inguinal swelling 8(16.7%) (Table 1).

All prostatic lesions were classified as benign and malignant .Out of 48 patients, 42 were of Benign Prostatic Hyperplasia and 6 were of adenocarcinoma.

Both BPH and adenocarcinoma were more prevalent in the age group of 60-70 years. Total 28 patients were in this age group, out of which 23 (85.2%) had BPH and 5 (17.9%) had Prostatic carcinoma (Table 2).

**Table 2:** Represent Correlation between Age groups, presenting complains and diagnosis

**Serial no**	**Variables**	**Diagnosis**	
		**Hyperplasia**	**Carcinoma**
1	**Age group**		
A	40-50	3 (100%)	0 (0%)
B	50-60	10 (90.9%)	1 (9.1%)
C	60-70	23 (85.2%)	5 (17.9%)
D	70-80	4 (100%)	0 (0%)
E	80-90	1 (100%)	0 (0%)
2	**Symptoms**		
A	Micturition	8 (80%)	2 (20%)
B	Abdominal pain	4 (100%)	0 (0%)
C	incontinence	8 (100%)	0 (0%)
D	Inguinal swelling	8 (100%)	0 (0%)
E	Urinary retention	21 (95.5%)	2 (8.7%)
F	Dribbling hematuria	10 (83.3%)	2 (16.7%)

Most common presenting symptoms of Benign Prostatic Hyperplasia were urinary retention 21(95.5%) and urinary dribbling 10(83.5%).

Patients of Prostatic carcinoma were mostly present with urinary retention 2(8.7%) and urinary dribbling 2(16.7%) (Table 2).

## Discussion

Two important histopathological prostatic lesions are benign prostatic hyperplasia and Prostatic carcinoma. These lesions cause enlargement of prostate gland, constricting the urethra and thus causing various urinary symptoms.

In our study, mean age of patients was 65.7 ± 7.6 years and mostly cases were present in the age group of 60-70 years (58.3%). These findings were similar with studies conducted in Pakistan, Oman, India and Saudi Arabia [6,11-13].

Most common prostatic lesion found in our study was Benign prostatic hyperplasia 42(87.5%) which was frequently found in the age group of 60-70 years. A study in India showed similar frequency of BPH (87%) and also same age group being affected [12]. A study in Nigerians and Saudi Arabia showed somewhat lower frequency (82%) but the peak age was similar with our study [13,14]. In our study. It was found that frequency of hyperplasia increases with age from fifth decade to seventh decade, this reflects that hyperplasia may be a normal aging process. It is believed that the main component of the hyperplastic process is impaired cell death due to which there is an overall reduction in the rate of cell death, resulting in the accumulation of senescent cells in the prostate. It is believed that DHT- induced growth factors cause increase proliferation of stromal cells and decreasing the death of epithelial cells. Microscopically hallmark of BPH is nodularity .Early nodules are composed of stromal cells and later predominantly epithelial nodules arise. From their origin, nodular enlargement encroaches into the lateral walls of urethra [10,15-17]. Study in Nigeria stated that incidence of BPH declines after 70 years of age same as stated in our study but notable book of Pathology “Pathologic basis of disease by Robbin and Cotrans “states that 70% of men suffers from BPH after their sixties and 90% after reaching at the age of 90% [10,14].

Commonest complains of patients of Benign prostatic hyperplasia were urinary retention 21 (95.5%) and, dribbling and hematuria 10 (83.3%). It may be due to the fact that increase size of gland and smooth muscle mediated obstruction of prostate cause urethral obstruction. Increased resistance to urinary flow leads to bladder hypertrophy and distention, accompanied by urine retention. Inability to empty the bladder completely creates a reservoir of residual urine that is a common source of infection, due to which urine frequency, nocturia, difficulty in starting and stopping of stream of urine ,overflow dribbling and dysuria occurs [10,18]. Bladder outflow obstruction may lead to Urinary Tract Infections, hydronephrosis and pyenephrosis.

After Benign Prostatic Hyperplasia, Prostatic Adenocarcinoma was found to be the commonest lesion (12.5%), occurs mostly in the same age group as BPH i.e. 60-70 years. Same findings were also reported in a study conducted in Faisalabad, Pakistan (13.2%) [6]. However, as compared to our study, in Oman, India and Saudi Arabia there was a slight lower incidence(10%) was seen [11-13]. Further, in contrary to our study, study in India showed peak age of incidence in the eight decade of life [12]. Androgen plays an important role in pathogenesis of prostate cancer. They bind to the androgen receptors and induce the expression of pro-survival and pro-growth. Men with germ line mutation of Tumor suppressor gene BRCA2 have a 20 fold increased risk of prostate cancer while the most common alteration in prostatic cancer is hypermethylation of glutathione S-trasferase (GSTP1) gene which down-regulates GSTP1 expression. Somatic mutations in prostate cancer gives rise to chromosomal rearrangements that juxtapose the coding sequence of an ETS family transcription factor gene (most commonly ERG or ETV1) next to the androgen –related TMPRSS2 promoter. Due to which ETS gene leads to over expression in androgen dependant fashion. Over expression makes normal prostate epithelial cells more invasive, possibly through the up regulation of matrix mettaloprotien. In addition, tumors which have rearranged ETS genes have certain distinctive morphologic features and have a different gene expression than those lacking ETS gene rearrangement [10].Dennis et al in his study had said that patients previously having prostitis are at risk of developing prostate cancer .Patients with prostatic carcinoma commonly presents with complains of micturition problems(20%), dribbling and hematuria (16.7%) ,and urinary retention (8.7%) as indicated in past study [19].

### Limitations

Cross-sectional study pattern was our biggest limitation, Second limitation was that our sample size didn’t represent the whole population; it was just based on the patients of civil hospital and Lyari General Hospital Karachi, Pakistan. Third limitation of our study was that we didn’t cover the aspects of family history, environmental factors, individual social class and ethnicity. We made all attempts to ensure that the data collected was reliable and the methods were reproducible. Our study opens the forum of discussion and should be continued in more advanced and modified phase. Thus, further prospective study on the basis of our findings will be highly recommended for the better understanding of relationship prostatic lesions with molecular pathogenesis and levels of androgens.

## Conclusion

Benign prostatic hyperplasia was the most common prostatic lesion occurring commonly after sixties with urinary retention having the most common complain. Screening protocols and awareness programs of Prostatic Cancer need to be introduced and screening programs should be focused on level of androgens and molecular pathogenesis.

## Competing interests

Authors declared that they have no competing interests.

## Authors’ contributions

HMA, NS, NAS did manuscript drafting while HAS SS and AM did data collection and critical review. All authors read and approved the final manuscript.
